# Interleukin 17A promotes diabetic kidney injury

**DOI:** 10.1038/s41598-019-38811-4

**Published:** 2019-02-19

**Authors:** Jin Ma, Yan J. Li, Xiaochen Chen, Tony Kwan, Steven J. Chadban, Huiling Wu

**Affiliations:** 1 Kidney Node Laboratory, The Charles Perkins Centre, Sydney Medical School, University of Sydney, Sydney, Australia; 2 Department of Renal Medicine, Royal Prince Alfred Hospital, Sydney, Australia

## Abstract

The role of the pro-inflammatory cytokine IL-17 in the pathogenesis of numerous inflammatory disorders is well-documented, but conflicting results are reported for its role in diabetic nephropathy. Here we examined the role of IL-17 signalling in a model of streptozotocin-induced diabetic nephropathy through IL-17 knockout mice, administration of neutralising monoclonal anti-IL-17 antibody and *in vitro* examination of gene expression of renal tubular cells and podocytes under high glucose conditions with or without recombinant IL-17. IL-17 deficient mice were protected against progression of diabetic nephropathy, exhibiting reduced albuminuria, glomerular damage, macrophage accumulation and renal fibrosis at 12 weeks and 24 weeks. Administration of anti-IL-17 monoclonal antibody to diabetic wild-type mice was similarly protective. IL-17 deficiency also attenuated up-regulation of pro-inflammatory and pro-fibrotic genes including IL-6, TNF-α, CCL2, CXCL10 and TGF-β in diabetic kidneys. *In vitro* co-stimulation with recombinant IL-17 and high glucose were synergistic in increasing the expression of pro-inflammatory genes in both cultured renal tubular cells and podocytes. We conclude that absence of IL-17 signalling is protective against streptozotocin-induced diabetic nephropathy, thus implying a pro-inflammatory role of IL-17 in its pathogenesis. Targeting the IL-17 axis may represent a novel therapeutic approach in the treatment of this disorder.

## Introduction

Diabetic nephropathy (DN) is now the leading cause of end-stage renal disease (ESRD) worldwide^1^. The rate of progression to ESRD in patients with diabetes and chronic kidney disease (CKD) has remained unchanged for decades, placing an enormous burden on healthcare systems^2^. Whilst recent developments demonstrating the reno-protective effect of sodium-glucose co-transporter 2 (SGLT2) inhibitors have provided some optimism, further insights into the pathogenesis of DN are required to facilitate future development of effective therapeutic strategies. Sterile inflammatory processes triggered by innate immune responses are known to contribute to DN development and progression^3,4^. IL-17A is an important regulator of innate immunity and has been implicated in the pathogenesis of several inflammatory diseases, but its role in CKD and specifically DN is less clear.

IL-17A is a member of the IL-17 family, which consist of six cytokines (IL-17A to IL-17F), of which IL-17A and IL-17F are the predominant isoforms. Members of the IL-17 family are traditionally considered potent pro-inflammatory cytokines primarily secreted by Th17 cells, but also produced by other cells including NK cells, macrophages neutrophils, dendritic and mast cells. There are five known receptors of the IL-17 family (IL-17RA through IL-17RE). IL-17A signals through the IL-17RA/IL-17RC complex^5–7^. IL-17RA and IL-17RC are found on the surface of many cell types including epithelial cells, fibroblasts, endothelial cells, astrocytes, macrophages and dendritic cells^5,6^. Upon activation by IL-17, IL-17Rs recruit Act1, triggering the NF-κB cascade resulting in the production of pro-inflammatory cytokines (IL-6, TNF-α, IL-1β), chemokines (CCL2 and CXCL2), and pro-fibrotic genes (TGF-β and fibronectin)^8,9^.

The pathogenicity of IL-17 has been well recognised in several diseases, including psoriasis^10^, rheumatoid arthritis^11^, multiple sclerosis^12^, cancer^13,14^ and diabetes^15–17^. Patients with diabetic retinopathy have elevated plasma IL-17 levels compared to healthy individuals^18^. Supportive evidence from rat models of Streptozotocin (STZ) induced diabetic retinopathy showed suppression with anti-IL-23, anti-IL-17A or anti-IL-17RA antibodies reduced diabetic retinal injury^19,20^. More recently, IL-17 has been associated with various kidney diseases^21^ including lupus nephritis^22–24^, ANCA-associated vasculitis^25–27^ and end-stage renal disease^28,29^. We have previously reported that IL-17A contributes to the development of kidney allograft rejection with IL-17A deficiency attenuating acute and chronic allograft injury, improving renal function and prolonging renal allograft survival^30^.

Current literature regarding the specific role of IL-17 in DN has been conflicting. Kim *et al*. reported attenuation of STZ-induced diabetic kidney injury by targeting Th17 cells through mycophenolate mofetil and concluded that modulation of IL-17 may be a viable therapeutic approach to treat DN^31^. This was corroborated in a diabetic rat model in which treatment with the mTOR inhibitor rapamycin attenuated Th17 activity and kidney injury^32^. Kuo *et al*. demonstrated infiltration of CD4^+^ IL-17^+^ T cells in human renal biopsies of both early and sclerotic DN, with both T cell infiltration and tissue IL-17A expression correlating with GFR decline^33^. In contrast, Mohamed *et al*. showed that IL-17A and IL-17F were protective against DN in mouse models^34^. In this study, diabetic IL-17^−/−^ mice displayed aggravated kidney damage compared to wild-type (WT) controls and administration of low-dose recombinant IL-17A was effective in the prevention and reversal of DN. This challenges the conventional notion of IL-17A as a pathogenic pro-inflammatory cytokine, but instead presents its role a modulator of inflammation. Therefore, further investigation to clarify the role of IL-17 in the pathogenesis of DN is required.

Here we examined the impact of IL-17 deficiency or blockade on the development of DN *in vivo*, and the effects of rIL-17 on kidney cells exposed to glucose *in vitro*, to demonstrate the involvement of IL-17A in DN and its role as a potential target for therapy.

## Results

### WT and IL-17^−/−^ mice developed equivalent STZ-induced diabetes

**Figure 1:**
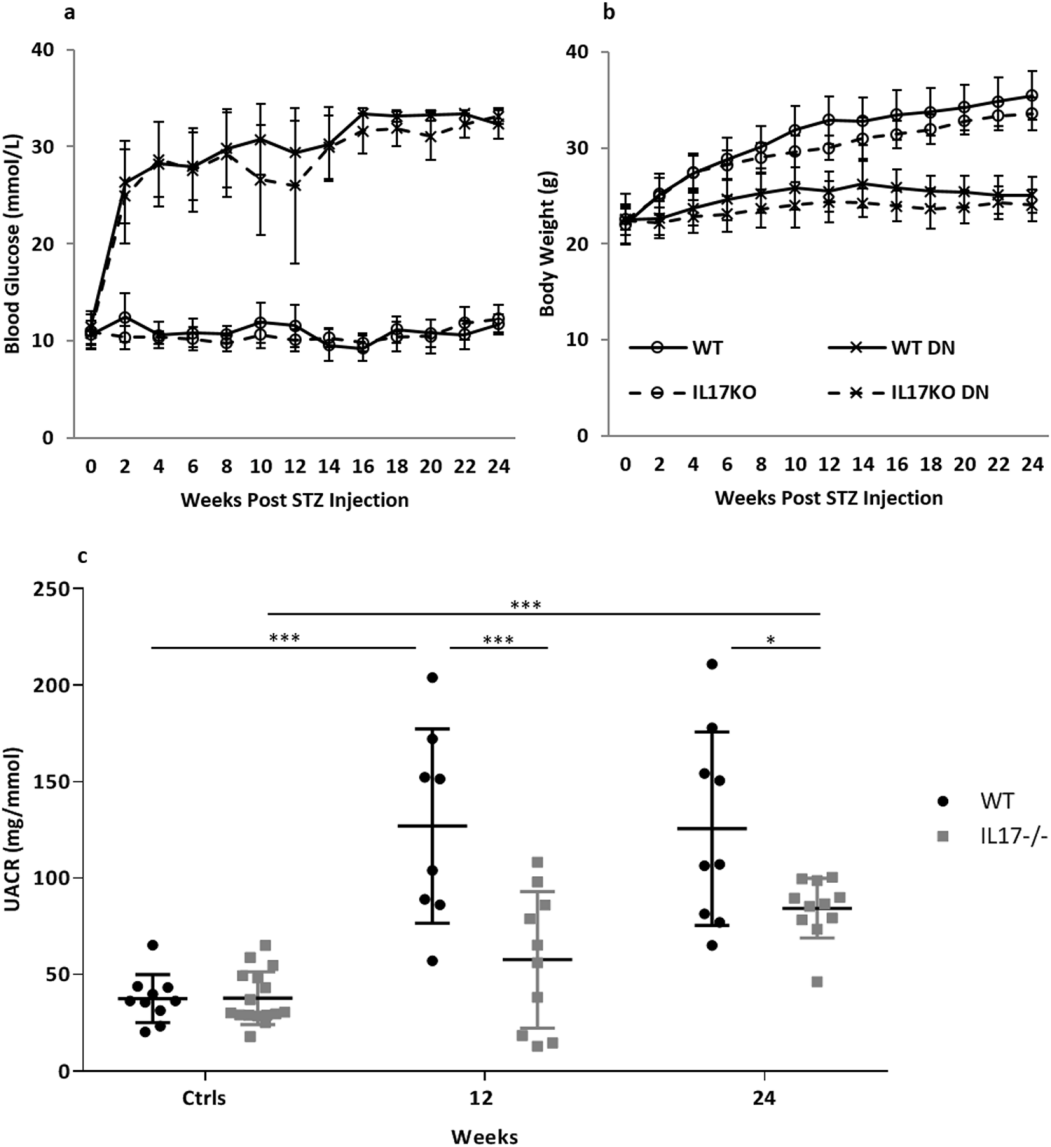
WT and IL-17^−/−^ mice developed equivalent degrees of hyperglycaemia, however IL-17 deficiency provides partial protection against albuminuria in DN. Streptozotocin (STZ) treatment induced diabetes in WT and IL-17^−/−^ mice with similar severity as indicated by blood glucose (**a**) and body weight (**b**) profiles over a period of 24 weeks. Progressive albuminuria is seen in diabetic mice compared to controls. However, diabetic IL-17^−/−^ mice develop significantly less albuminuria than WT mice, with no significant difference compared to non-diabetic controls until week 24 (**c**). Data are shown as means ± SD; *P < 0.05; ***P < 0.001. WT n = 8 and IL-17^−/−^ n = 10 for week 12 time point and WT n = 9 and IL-17^−/−^ n = 11 for week 24 time point in diabetic groups. Age matched non-diabetic controls n = 5 mice per group at 12 weeks, WT n = 5 and IL-17^−/−^ n = 11 for week 24. Data are shown as means ± SD.

WT and IL-17^−/−^ mice treated with STZ displayed a similar profile in the progression of hyperglycaemia (Fig. 1a) and weight gain (Fig. 1b) over a 24 week period.

### IL-17 deficiency attenuates albuminuria

Whilst both diabetic WT and IL-17^−/−^ mice developed albuminuria within 24 weeks of diabetes induction compared to their non-diabetic controls (Fig. 1c), diabetic IL-17^−/−^ mice exhibited significantly reduced albuminuria compare to diabetic WT mice at weeks 12 (127.1 vs 57.9 mg/mmol, *p* < 0.001) and 24 (125.7 vs 87.3 mg/mmol, *p* < 0.05).

### IL-17 deficiency reduced kidney glomerular injury and hypertrophy

**Figure 2:**
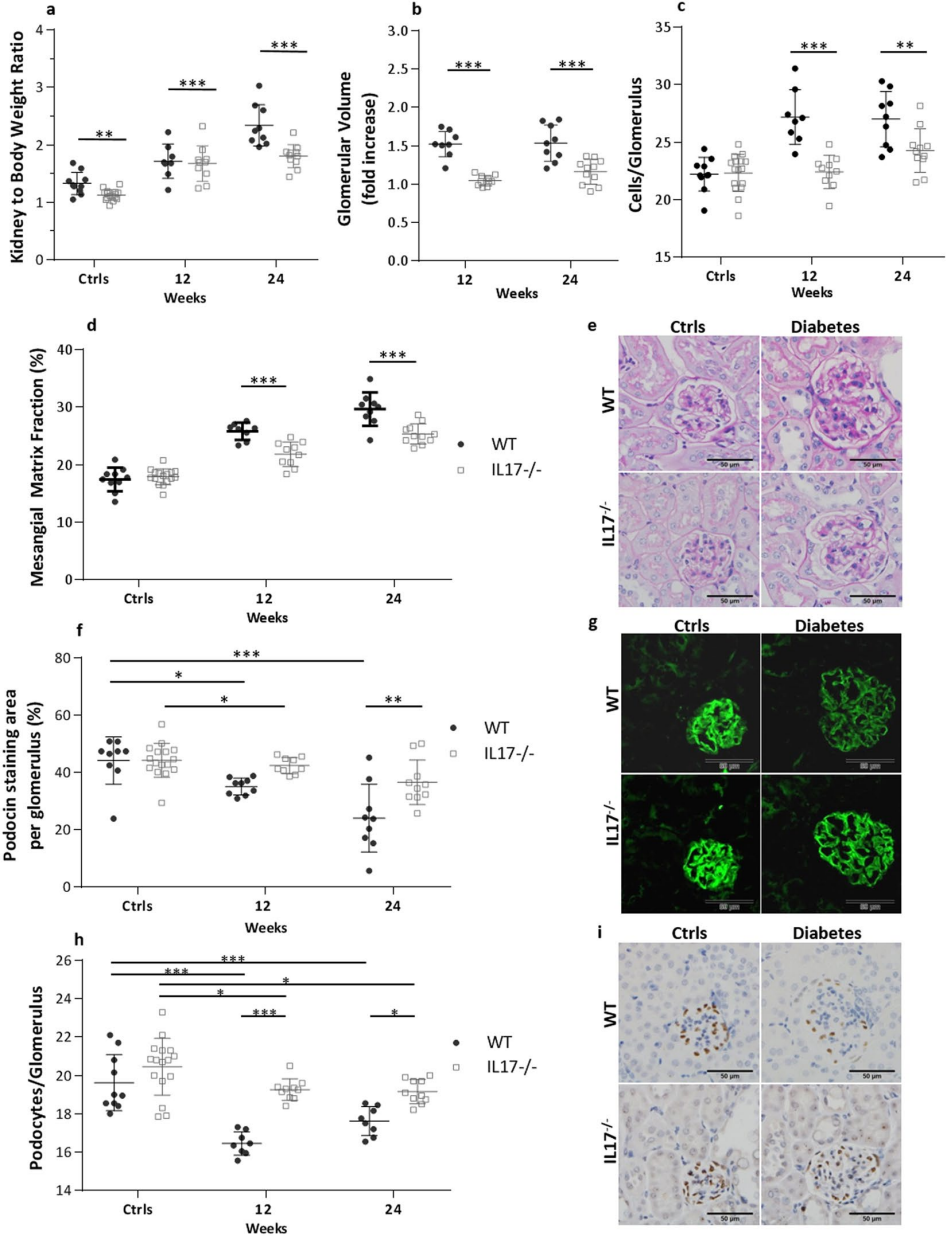
IL-17 deficiency reduces glomerular and interstitial injury in DN. Diabetic IL-17^−/−^ mice are relatively protected from glomerular and interstitial injury from DN compared to diabetic WT mice, as indicated by decreased kidney to bodyweight ratio (**a**) glomerular volume (**b**,**e**), glomerular hyper-cellularity (**c**,**e**) and mesangial expansion (**d**,**e**). Podocyte damage, assessed by podocin staining is also less severe in diabetic IL-17^−/−^ mice compared to WT diabetic mice (**f**,**g**). (**e**) Representative sections of glomeruli from WT and IL-17^−/−^, diabetic and non-diabetic mice at 24 weeks (PAS stained, ×400 magnification). (**f**) Representative sections of glomeruli stained for podocin at 24 weeks (×400 magnification), demonstrating similar staining intensity in non-diabetic WT and IL-17^−/−^ mice, reduced staining in diabetic mice, with more pronounced reduction in WT versus IL-17^−/−^ diabetic mice. Data are shown as means ± SD; ***P < 0.001, **P < 0.01, *P < 0.05. The number of animals per group was defined in Fig. 1.

Diabetic WT mice developed progressive kidney hypertrophy as shown by 30% and 76% increase of kidney to body weight ratio at week 12 and 24 respectively, compared to their non-diabetic WT controls (Fig. 2a). The diabetic IL-17^−/−^ mice developed similar kidney hypertrophy at 12 weeks however progression was attenuated by 24 weeks compared to diabetic WT mice. Diabetic WT mice demonstrated significantly increased glomerular volume compared to non-diabetic WT controls at both 12 and 24 weeks, while this was significantly reduced in diabetic IL-17^−/−^ mice compared to diabetic WT mice at both time points (Fig. 2b,e). Glomerular hypercellularity was evident at 12 and 24 weeks in diabetic WT kidneys, whilst significantly diminished in diabetic IL-17^−/−^ kidneys at both time points (Fig. 2c). Computerised morphometric analysis of PAS-stained kidney sections revealed significant mesangial expansion in diabetic WT glomeruli, which was attenuated in diabetic IL-17^−/−^ glomeruli (Fig. 2d,e). To evaluate podocyte injury, which typically correlates with albuminuria in DN, we assessed protein expression of the podocyte markers podocin and WT1 by immunofluorescence and immunohistochemistry, respectively. Diabetic WT kidneys displayed a progressive reduction of podocin expression compared to non-diabetic WT controls over the experimental time course (Fig. 2f,g). By comparison, loss of podocin staining was less pronounced in diabetic IL-17^−/−^ kidneys compared to diabetic WT kidneys at 24 weeks. A reduction in the number of WT1^+^ cells was seen in diabetic WT kidneys compared to their non-diabetic controls at 12 and 24 weeks. However, loss of WT1^+^ cells in diabetic IL-17^−/−^ kidneys was significantly reduced compared to diabetic WT kidneys at weeks 12 and 24 (Fig. 2h,i).

### IL-17 deficiency protected diabetic kidneys from fibrosis

**Figure 3:**
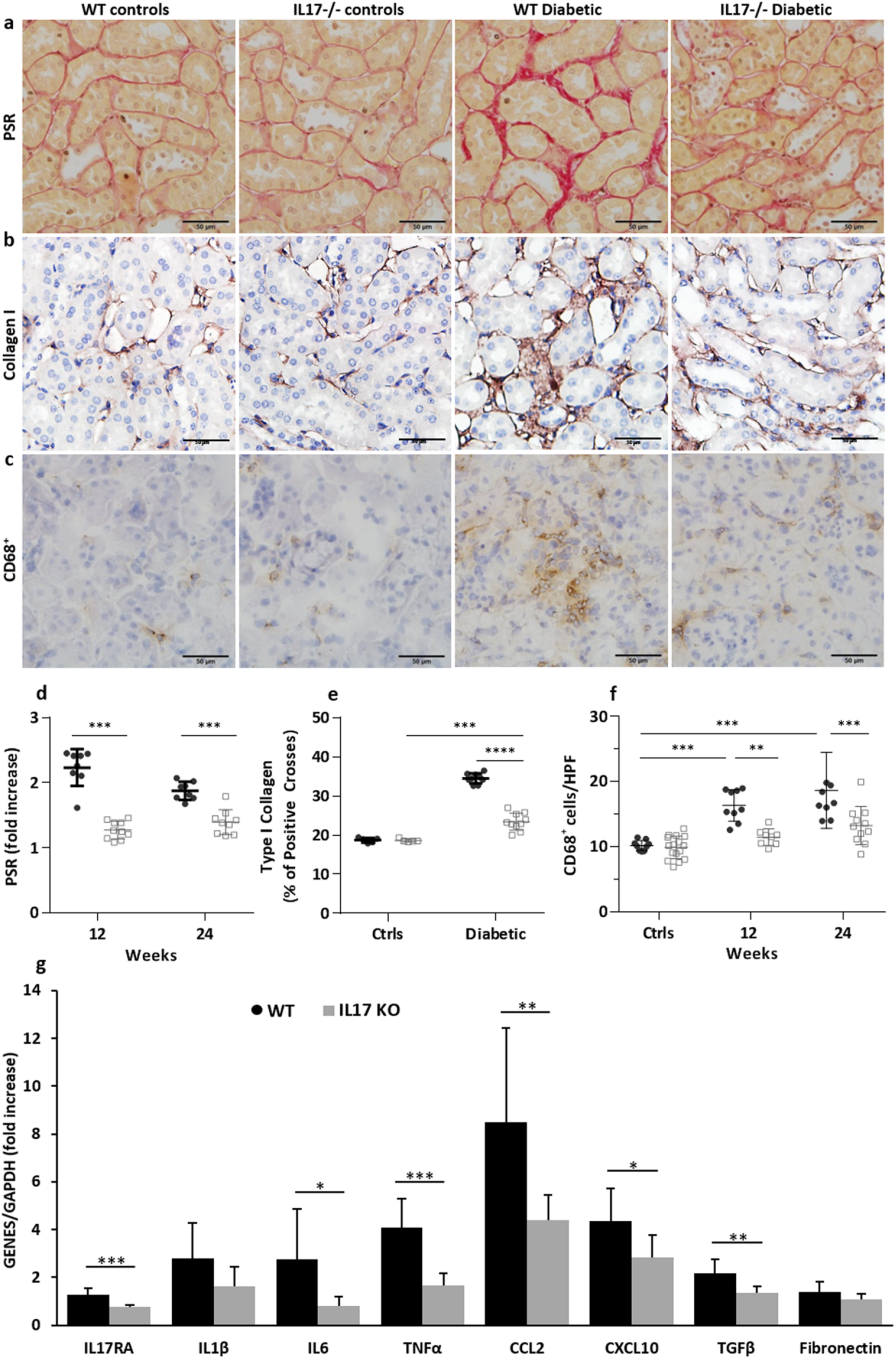
IL-17 deficiency protects diabetic kidneys from interstitial fibrosis, delays macrophage accumulation and reduces pro-inflammatory and pro-fibrotic gene expression. Increased interstitial collagen accumulation was evident in WT compared to IL-17^−/−^ diabetic mice at both 12 and 24 weeks (**d**,**e**). Representative sections of kidney from WT and IL-17^−/−^ diabetic and nondiabetic mice at 24 weeks demonstrating the increased interstitial collagen deposition in WT diabetic mice being attenuated by IL-17 deficiency using PSR staining (**a**) and immunostaining for Collagen I (**b**). Interstitial CD68 + macrophage accumulation is also evident in diabetic WT but not IL-17 deficient mice, until a mild increase appears at week 24 (**f**). (**c**) Representative sections of kidney from WT and IL-17^−/−^, diabetic and non-diabetic mice at 24 weeks stained for CD68. (**g**) RT-PCR demonstrates substantial up-regulation of mRNA expression of IL6, TNF-α, CCL2, CXCL10 and TGF-β in WT diabetic kidneys at 12 weeks, all of which are diminished in the setting of IL-17 deficiency. Data are shown as means ± SD; ***P < 0.001, **P < 0.01, *P < 0.05. The number of animals per group was defined in Fig. 1.

The degree of interstitial fibrosis is a strong indicator of progression to kidney failure. Diabetic WT kidneys demonstrated significantly more interstitial fibrosis than non-diabetic WT kidneys at 12 and 24 weeks. These changes were significantly diminished in diabetic IL-17^−/−^ kidneys compared to diabetic WT group. Quantification of interstitial fibrosis using Picro-Sirius Red (PSR) and immunohistochemical staining for Type I Collagen (Col-1) revealed substantial interstitial collagen deposition in diabetic WT kidneys compared to non-diabetic WT kidneys. Compared to diabetic WT kidneys, diabetic IL-17^−/−^ kidneys demonstrate a significant reduction in interstitial collagen deposition at 12 and 24 weeks, respectively (*p* < 0.001), (Fig. 3a,b).

### IL-17 deficiency attenuated macrophage accumulation in diabetic kidneys

Immunostaining of a pan-macrophage marker, CD68 indicated a dramatically increased number of interstitial macrophages at weeks 12 and 24 in diabetic WT kidneys compared to non-diabetic WT kidneys (Fig. 3c,f). This accumulation of CD68 positive cells was reduced in diabetic IL-17^−/−^ kidneys at week 12 and 24 compared to diabetic WT groups.

### IL-17 deficiency suppressed expression of pro-inflammatory and pro-fibrotic genes in diabetic kidneys

Gene expression of inflammatory cytokines, chemokines and fibrosis-related genes in the kidney were examined by real-time PCR (Fig. 3g). IL-6 and TNF-α mRNA expression were up-regulated in WT diabetic kidneys compared with non-diabetic WT controls. In contrast, the up-regulation of IL-6 and TNF-α were not observed in diabetic IL-17^−/−^ kidneys. Diabetic WT kidneys also exhibit substantial upregulation of the chemokines CCL2 and CXCL10 compared to non-diabetic WT controls. Chemokine expression was attenuated in diabetic IL-17^−/−^ kidneys compared to the diabetic WT group. With regards to fibrosis-related genes, upregulation of TGF-β in diabetic kidneys was attenuated in IL-17^−/−^ mice compared to diabetic WT mice, but fibronectin expression remained equivocal.

### Treatment with neutralising antibody to IL-17 is also protective against DN

**Figure 4:**
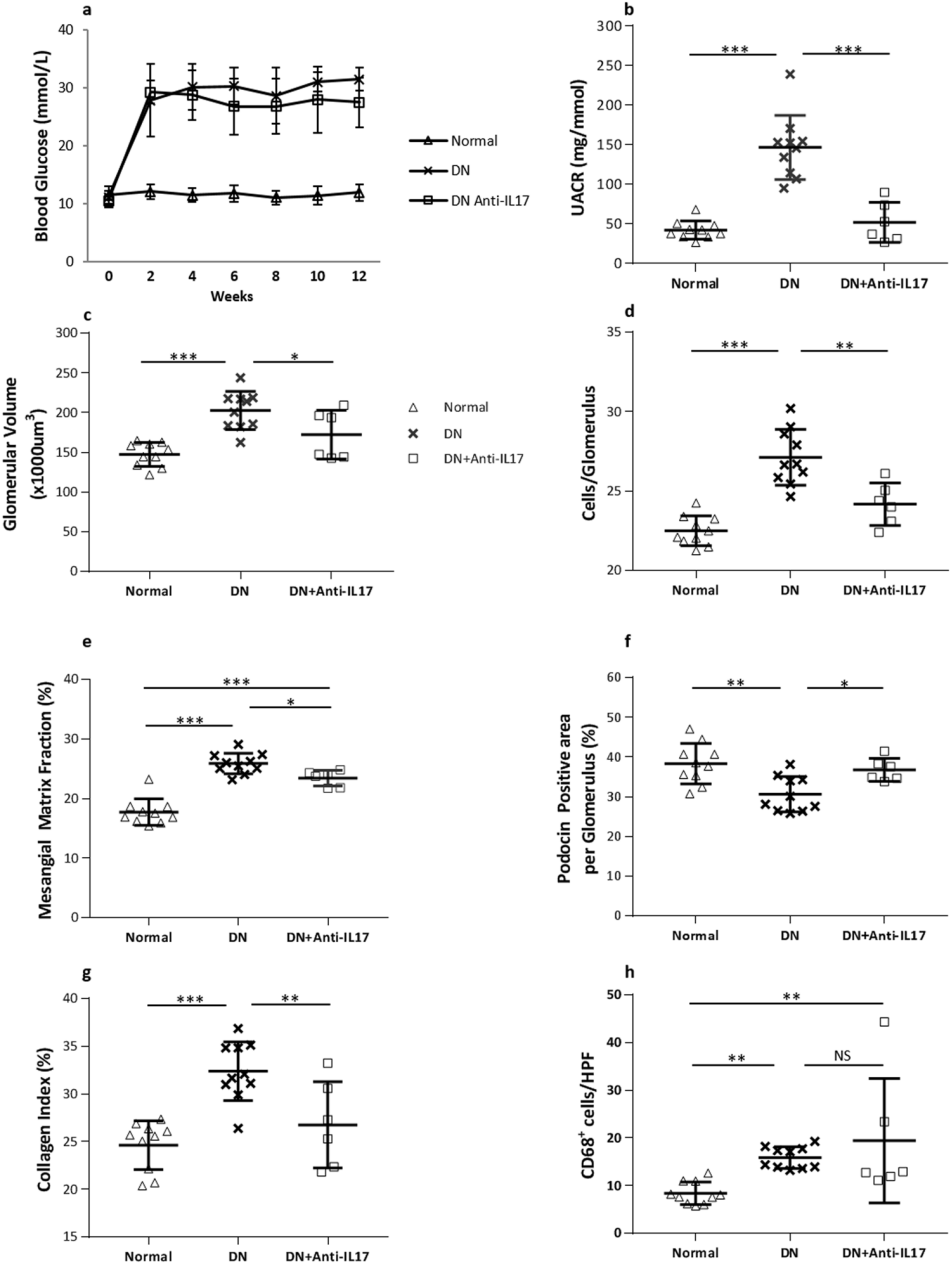
Administration of neutralizing IL-17 antibody to WT diabetic mice ameliorates diabetic kidney injury. Anti-IL-17 antibody treatment (200 μg per animal, twice a week, starting at 3 weeks post first STZ injection) has no impact on blood glucose levels (**a**), whilst albuminuria is significantly attenuated at week 12 to a level comparable to that of IL-17^−/−^ control mice (**b**). Diabetic glomerular and interstitial changes including glomerular volume (**c**), hyper-cellularity (**d**), mesangial expansion (**e**), podocyte injury (**f**) and interstitial fibrosis (**g**) are diminished with anti-IL-17 antibody treated vs. non-treated diabetic mice. However, anti-IL-17 antibody treatment does not prevent renal accumulation of CD68 + positive cells (**h**). Data are present as means ± SD. *P < 0.05, **P < 0.01, ***P < 0.001. n = 10 for non-diabetic and diabetic groups, n = 6 for anti-IL-17 antibody treated group.

The protection against DN conferred by IL-17 deficiency in knockout mice prompted us to assess the efficacy of a more clinically relevant strategy to IL-17 blockade via administration of neutralising anti-IL-17 Ab. Treatment of WT mice with anti-IL-17 Ab after the development of diabetes was also renoprotective. Despite developing a similar degree of hyperglycaemia to untreated diabetic WT controls (Fig. 4a), anti-IL-17 Ab treatment significantly attenuated albuminuria at 12 weeks (Fig. 4b). Consistent with this, reductions in histologic parameters of DN including glomerular hypertrophy, hypercellularity, podocyte loss, mesangial expansion and interstitial collagen deposition were observed as shown in Fig. 4c–g. No significant difference of CD68^+^ macrophage accumulation in diabetic kidneys was observed in anti-IL-17 Ab treated group compared to WT controls (Fig. 4h).

### IL-17A synergises with high glucose to promote inflammation, but not fibrosis in primarily cultured TECs and podocytes

To confirm whether IL-17 exacerbates the pathological changes in kidney cells under hyperglycaemic conditions, primary tubular epithelial cells (TEC) and podocyte cultures were stimulated with rIL-17 under normal or high glucose conditions.

**Figure 5:**
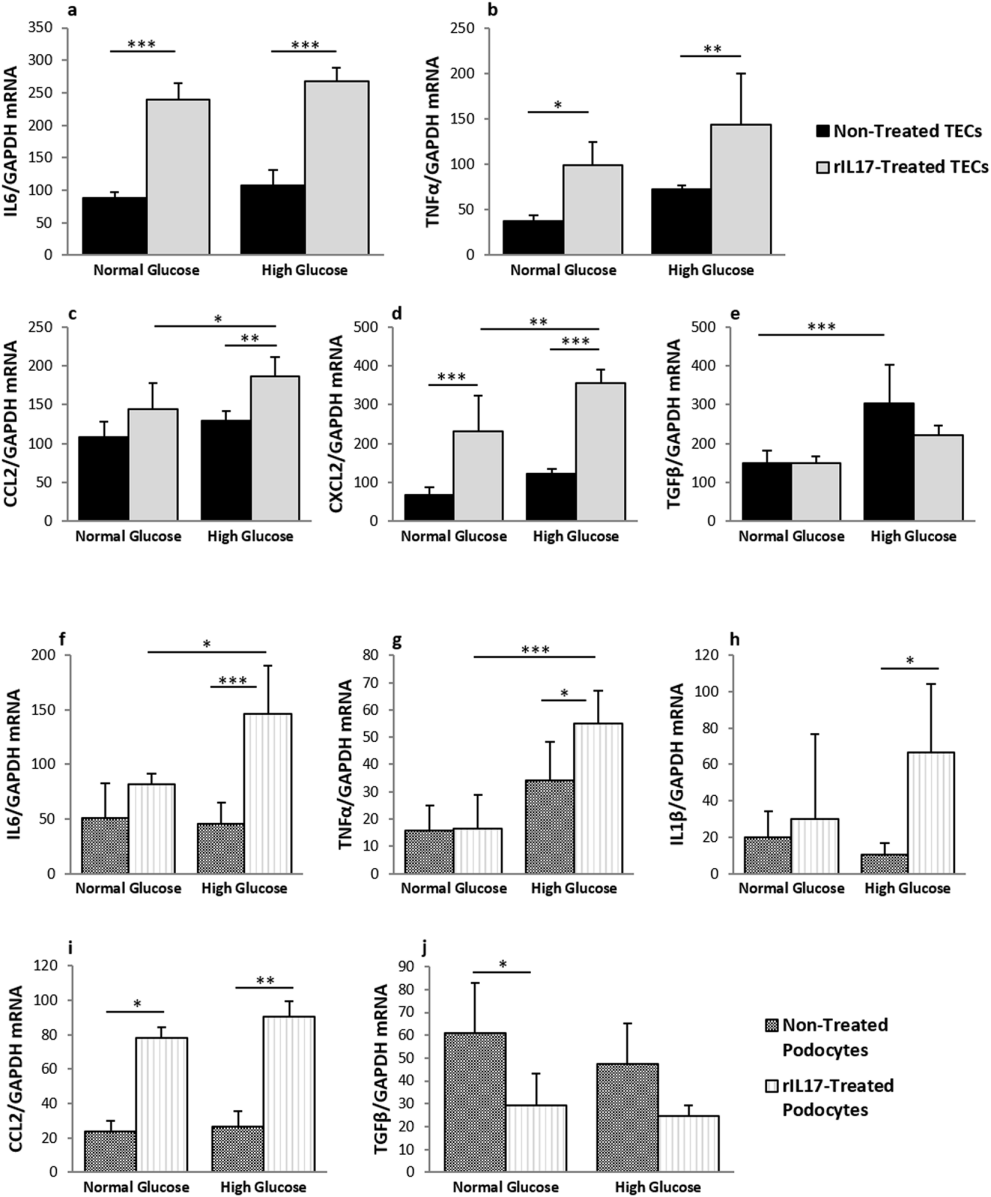
IL-17 and high glucose synergistically promote inflammation in cultured tubular epithelial cells (TEC) and podocytes. TECs or podocytes are stimulated with high glucose (5.5 mM glucose + 24.5 mM glucose) or recombinant IL-17 (rIL-17) (100 ng/ml) alone or in combination for 24 hours. In the normal glucose groups, 24.5 mM mannitol is added to control osmotic pressure. TECs express higher level of TNFα (**b**) CCL2 (**c**) and CXCL2 (**d**) after co-stimulation with both high glucose and rIL-17, compared to stimulation with either effector alone, whilst IL-6 expression only responds to rIL-17 stimulation (**a**). rIL-17 did not alter TEC TGFβ expression under either normal or high glucose conditions (**e**). In primarily cultured podocytes IL-6 (**f**) and TNFα (**g**) are up-regulated significantly by the combination of rIL-17 and high glucose, but not by rIL-17 or high glucose alone. IL-1β expression is upregulated by rIL-17 under a high, but not normal glucose condition (**h**). rIL-17 alone up-regulated CCL2 expression, with additional high glucose not providing any further effect (**i**). In contrast, rIL-17 downregulates TGF-β expression in the presence of high glucose (**j**). Data are shown as means ± SD; *P < 0.05, **P < 0.001, ***P < 0.0001 (n = 6).

In the primary TEC cultures, rIL-17 stimulation significantly upregulated the expression of IL6 and TNFα cytokines under normal glucose conditions, and the presence of high levels of glucose did not provided additional effect (Fig. 5a,b). Upregulation of CCL2 chemokine by rIL-17 required high glucose conditions, while rIL-17 or high glucose alone was not sufficient for the upregulation of CCL2 (Fig. 5c). Additionally, rIL-17 upregulated the expression of CXCL2, and was further enhanced under high glucose conditions (Fig. 5d). rIL-17 did not alter TGFβ expression under either normal or high glucose conditions (Fig. 5e).

In podocyte cultures, IL-6 (Fig. 5f) and TNF-α (Fig. 5g) were upregulated by rIL-17, and these upregulations were further exaggerated by the presence of high glucose. IL-1β expression was also upregulated by rIL-17 under a high, but not normal glucose condition (Fig. 5h). rIL-17 upregulated CCL2 expression in podocytes with or without the presence of high glucose (Fig. 5i). rIL-17 downregulated TGF-β expression with the presence of high glucose (Fig. 5j).

## Discussion

IL-17A is a pleiotropic cytokine implicated in disease processes by promoting inflammation through induction of chemokine expression, pro-inflammatory cytokines and matrix metalloproteases. Yet recent studies suggest a more complex and often paradoxical role for IL-17 in kidney disease^35–37^. Our study provides further insights into the role of IL-17 in diabetic kidney disease. We observed that genetic depletion of IL-17 did not alter the blood glucose profile in diabetic mice, but did attenuate albuminuria, glomerular hypertrophy, interstitial fibrosis and inflammation within the kidney, suggesting a pathogenic role for IL-17A in a multi-low dose STZ-induced model of DN. Similar beneficial effects were achieved by administration of a neutralising IL-17A antibody to diabetic WT mice, indicating the favourable alterations seen in IL-17A^−/−^ diabetic mice are specific to IL-17A deficiency.

Albuminuria is both an early marker of DN and reflective of podocyte injury. In DN, reduction in podocyte number correlates with the degree of albuminuria, GFR decline and is a strong predictor of eventual disease progression^38^. Studies have linked IL-17 signalling with promotion of podocyte injury in both primary nephrotic syndrome and adriamycin-induced nephropathy^39,40^. Stimulation of podocytes with rIL-17 *in vitro* induced inflammation and apoptosis through secretion of IL-1β and activation of the NLRP3 inflammasome^41^. In our study, primary cultures of podocytes displayed up-regulated expression of pro-inflammatory cytokines and chemokines in response to high glucose conditions. Furthermore, stimulation with both rIL-17 and high glucose was more effective in increasing the expression of inflammatory cytokines IL-6 and TNFα and the chemokine CCL2 than either condition alone, suggesting IL-17 and hyperglycaemia synergistically promote diabetic podocyte injury. This is supported by our *in vivo* observation of decreased albuminuria in IL-17^−/−^ diabetic mice compared to WT diabetic mice, with decreased podocyte injury demonstrated on immunostaining for the podocyte markers WT1 and podocin. Taken together, these findings implicate a role for IL-17 in diabetic podocytopathy.

DN is also characterised histologically by glomerular basement membrane thickening and mesangial expansion. We found depletion of IL-17 by either gene deletion or neutralising antibody administration attenuated mesangial expansion in diabetic kidneys. Hyperglycaemia and advanced glycation end products (AGEs) are known to stimulate mesangial cells to proliferate and produce extracellular matrix through chemokine signalling in DN^42,43^. Interestingly, IL-17 has also been shown to increase mesangial expression of IL-17Rs and downstream pro-inflammatory chemokine expression including CCL2^44,45^. This upregulation of chemokines in mesangial cells is known to be critical in renal leukocyte recruitment and mesangial matrix expansion, with therapeutic blockade of CCL2 in murine models reducing collagen matrix fraction and macrophage infiltration^46^. Macrophage infiltration itself is associated with progression of DN in human and animal models^47,48^. Under high glucose conditions *in vitro*, renal tubular epithelial cells have been shown to increase the expression of IL-17 together with TGF-β1 and IL-6^49^. IL-17 enhances the up-regulation of IL-6, TNF-α and CCL2 in mesangial and tubular epithelial cells resulting in local macrophage recruitment^44,45,50^. Our immunohistological data reflects this, with reduced interstitial macrophage infiltration in the diabetic IL-17^−/−^ kidneys compared to WT kidneys. The expression of above inflammatory cytokines and chemokines in renal tissue was also diminished by IL-17 deficiency. However whilst macrophages are traditionally thought of as effectors of injury in DN, the balance of pro-inflammatory (M1) and anti-inflammatory (M2) macrophages is important. Adoptive transfer of M2 polarised macrophages into diabetic mice has been shown to result in gradual renal accumulation, which was protective against both renal injury and fibrosis^51^. We found that whilst rIL-17 antibody administration to diabetic mice reduced renal injury, this treatment exhibited no impact on macrophage infiltration compared to non-treated mice. This effect may represent a shift along the M1/M2 spectrum, with alternatively activated macrophages tilting this balance. Finally, we demonstrated that under high glucose conditions, administration of rIL-17 provided additional effect in augmenting inflammation in cultured tubular epithelial cells and podocytes, suggesting that IL-17 plays a specific role in hyperglycaemic conditions to promote local inflammation and accelerate progression of DN.

The critical role of tubulo-interstitial fibrosis in the progression of DN to ESRD has been well recognised^52^. IL-17 participates in a positive feedback loop with IL-6, inducing the activation of NF-κB signalling which results in subsequent overexpression of various chemokines, fibrotic genes including TGF-β, and engagement of signal transducer and activator of transcription 3 (STAT3)^53,54^. STATs may also promote tissue fibrosis by mediating overexpression of TGF-β^55^. Diabetic IL-17^−/−^ mice in our study had substantially reduced expressions of IL-6, TGF-β and collagen deposition in the kidney than evident in diabetic WT mice. Interestingly, the pro-fibrotic effect of IL-17 we saw on whole kidney tissue *in vivo* was not reproduced by our *in vitro* study of TECs and podocytes. Notably in podocytes, the presence of IL-17 even appeared to suppress the expression of TGF-β. This may reflect that *in vitro* conditions do not fully recapitulate the events of *in vivo* systems. Yet Sun *et al*. have demonstrated cell-origin-dependent effects of IL-17 on fibrosis, with inhibitory effects on renal fibroblasts, increased activation in pulmonary fibroblasts, and no effect on foetal fibroblasts^35^. Thus, the possibility of IL-17 exerting differential effects in a tissue and cell-specific manner cannot be discounted.

The complex role of IL-17 is further highlighted by a recent report from Mohamed *et al*.^34^. In their study, IL-17^−/−^ mice exhibited more severe diabetic kidney injury compared to WT mice, with administration of low doses of rIL-17A or F being protective against DN. In contrast to our multi-low-dose STZ diabetic model, Mohamed *et al*. used a single high dose of STZ to induce diabetes^34^. The potential for collateral tissue toxicity caused by high dose STZ may confound both the clinical context and interpretation of renal injury^56^. Although the authors’ demonstrated administration of rIL-17 ultimately attenuated diabetic kidney injury in their models, the specific cell types influenced by IL-17 were not investigated. Our *in vitro* studies show rIL-17 suppressed TGF-β expression in cultured primary podocytes, raising the possibility that podocytes maybe the cell responsible for modulating their demonstrated protective effects of IL-17 in DN. This negative association between IL-17 and TGF-β was also reported in a clinical study of metabolic syndrome^57^. To confirm such a hypothesis, mice with cell-specific knockdown of IL-17 in podocytes and/or tubular epithelial cells will be required^36^.

In conclusion, our study demonstrates a pro-inflammatory role for IL-17 in mediating podocyte injury, mesangial expansion and renal fibrosis in DN. Although recent studies have challenged the traditional pathogenic role of IL-17, our results further highlight the complexity of immune mechanisms in diabetic kidney damage. Targeting the IL-17 axis may represent a novel therapeutic approach, however clarifying the specific conditions under which IL-17 exacerbate or attenuate DN warrant further investigation.

## Methods

### Animals

Male Wild-type (WT) C57BL/6 mice were obtained from the Animal Resource Centre (Perth, Australia). IL-17 deficient mice on a C57BL/6 background were provided by Professor Geoffrey Hill (Queensland Institute of Medical Research, Brisbane, Australia), with approval from Professor Yoichiro Iwakura (Centre for Experimental Medicine, The University of Tokyo, Japan). The mice were housed in a specific pathogen-free facility at the University of Sydney. Male mice aged 8-9 weeks were used in all experiments. All animal experiments were performed with the approval of the animal ethics committee of the University of Sydney. The methods were carried out in accordance with the approved guidelines and regulations.

### Induction of diabetes

Male WT and IL-17^−/−^ mice were fasted for 4 hours before administration of intraperitoneal streptozotocin (STZ, Sigma-Aldrich) at a dose of 55 mg/kg for 5 consecutive days. Citrate buffer was used as a vehicle control. Mice with a blood glucose level over 20 mmol/L were used for assessing diabetic kidney injury. Animals were sacrificed 12 (WT n = 10; IL-17A^−/−^ n = 12) and 24 (WT n = 10; IL-17A^−/−^ n = 11) weeks after STZ injection. There were 5–10 mice per control group.

### IL-17 antibody treatment

Three weeks after initial STZ administration, once hyperglycemia was well established, a group of WT diabetic mice (n = 7) were given intraperitoneal neutralising IL-17A monoclonal antibody (clone 17F3, BioXCell) at a dose of 0.2 mg per animal, twice weekly until week 12.

### Sample collection

Mice were placed in metabolic cages for 16 hours prior to sacrifice for collection of urine. Blood and tissues samples were harvested at sacrifice. In brief, 1 mL of blood was harvested via intracardiac puncture and processed for serum. The spleen, pancreas and kidney were harvested. Tissue slices were fixed with 10% neutral-buffered formalin for paraffin embedding, frozen in OCT compound (Sakura Finetek Inc., Torrance, CA) or snap frozen in liquid nitrogen for mRNA extraction.

### Quantification of albuminuria and urine creatinine

Urine albumin was quantified using the Mouse Albumin ELISA Quantitation Set (Bethyl Laboratories, Montgomery, TX, USA) as described previously^58^. Urine creatinine was measured enzymatically by the Biochemistry Department of Royal Prince Alfred Hospital, Sydney, Australia.

### Real-time RT-PCR

Total RNA was extracted using TRIzol (Invitrogen). cDNA was synthesised using oligo(dT)_16_ primers (Applied Biosystems, Foster City, CA) and the SuperScript III reverse transcriptase kit (Invitrogen) according to the manufacturer’s instructions. cDNA was amplified in Universal Master Mix (Applied Biosystems) with gene-specific primers and probes, using the Rotor-Gene 6000 system (Corbett Life Science). Specific TaqMan primers and probes for IL-6, TNFα, CCL2, CXCL10, TGF-β1, fibronectin, and GAPDH have been described previously^58,59^. Taqman primers and probes for IL-17A (Mm00439619_m1) and IL-17RA (Mm00434214_m1) were obtained from Applied Biosystems. All expressed results were normalised to GAPDH expression.

### Histology

Periodic acid–Schiff’s (PAS) and Picro-Sirius Red (PSR) staining were performed on 3 µm and 5 µm formalin-fixed kidney sections, respectively. Glomerular tuft area (A_G_) was measured by microscopy using DP2-BSW software (V2.2, OLYMPUS). Mean glomerular volume (V_G_) was calculated using the formula described by Weibel and Gomez^60^; V_G_ = (β/k) × (A_G_)^3/2^, where k = 1.1 (size distribution coefficient) and β = 1.38 (shape coefficient for spheres). In each glomerular tuft, mesangial area was defined as positive staining with PAS and enumerated by image analysis software (Image Pro Premier 9.0, Media Cybernetics), expressed as percentage of total glomerular area^61^. Total glomerular cellularity was determined by tallying nuclei in glomerular cross-sections using ImageJ software. Interstitial collagen on PSR-stained sections were assessed by point counting using an ocular grid as described by McWhinnie *et al*.^62^ in at least 20 consecutive fields (×400 magnification). Only interstitial collagen was counted, with vessels and glomeruli excluded. The results were expressed as the percentage of positive staining points per field.

### Immunohistochemistry

Staining for WT1 and CD68 were performed on acetone-fixed frozen sections (7μm) after endogenous biotin was blocked using a biotin blocker system (DAKO, Carpinteria, CA). To detect Type 1 Collagen (Col-1), formalin-fixed sections (5μm) were deparaffinised and antigen retrieval performed by boiling sections for 10 minutes in 10 mM sodium citrate buffer (pH 6.0). Sections were then incubated with 10% normal horse serum followed by 60 minute incubation with primary antibodies: rat anti-mouse CD68 (ABD Serotec Inc., Oxford, UK), rabbit anti-WT1 (Abcam, Cambridge, UK), rabbit anti-Col-1 (Abcam, Cambridge, UK) or concentration-matched isotype negative control. Endogenous peroxidase activity in the sections was quenched with H_2_O_2_ prior to application of biotinylated anti-rat IgG or anti-rabbit IgG (BD Biosciences, Pharmingen). A Vector stain ABC kit (Vector Laboratories Inc) was applied to the tissue followed by DAB solution (DAKO).

### Immunofluorescence

Podocin staining was performed on 7μm acetone-fixed frozen sections. After blocking with 10% normal horse serum, sections were incubated with a rabbit anti-NPHS2 antibody (Abcam) at 4 °C overnight. For detection, sections were incubated with an Alexa Fluor® 488-conjugated anti-rabbit antibody for 1 hour.

### Quantification of immunostaining

Glomerular CD68 and WT1^+^ cells were counted in glomerular cross-sections (×400 magnification). Analysis of interstitial CD68^+^ cells was performed by assessing twenty consecutive high-power fields (HPFs; magnification, ×400) of renal cortex in each section. Using an ocular grid, the number of cells staining positively for each antibody was counted and expressed as cells per field. Podocin expression was assessed in glomerular cross-sections using Image Pro. The lower threshold for positive glomerular staining was determined by the highest background fluorescence in the non-glomerular area of each section. Results were expressed as a percentage of positive staining per glomerulus^58,63^. Interstitial Col-1 was assessed by point counting using an ocular grid in 20 consecutive fields (×400 magnification) excluding vessels and glomeruli. Results were expressed as the percentage of positive staining points per field.

### Primary culture of podocytes

Podocytes were isolated and cultured as described previously^64^. Briefly, the kidneys were perfused with 10^7^ Dynabeads and the cortex was cut into small pieces (1–2 mm^3^) and digested in 2 mg/mL collagenase at 37 °C for 30 min. The collagenase-digested tissue was passed through a 100 µm sieve and centrifuged at 200 *g*. The pellet was resuspended and glomeruli-containing Dynabeads were gathered in a magnetic field. The glomeruli were pipetted onto a 40 µm nylon sieve to remove free Dynabeads and collected by washing through an inverted nylon sieve.

Isolated glomeruli were seeded onto collagen-coated culture dishes (BD Biosciences) in DMEM/F-12 medium containing 5% foetal bovine serum supplemented with 0.5% insulin-transferrin-sodium selenite (ITSS), 100 U/mL penicillin and 100 mg/mL streptomycin (Invitrogen) and incubated at 37 °C. The cultured cells were examined for the podocyte markers podocin and nephrin by immunofluroesent staining. Cells were >95% positive for these markers. Experiments were commenced after cells had reached 80% confluence.

### Primary culture of mouse tubular epithelial cells (TEC)

Mouse kidney TECs were isolated and cultured as described previously^65^. In brief kidneys were perfused with saline then removed. Kidney cortices were dissected into approximately 1 mm^3^ pieces and digested in HBSS containing 3 mg/mL of collagenase at 37 °C for 25 minutes, followed by washing in DMEM/F12 medium (Invitrogen). The kidney digest was washed through a series of sieves (mesh diameters of 250, 150, 75 and 40 µm) then spun down at 300 g for 5 minutes. The cell pellet was re-suspended in defined K1 medium: DMEM/F12 supplemented with 25 ng/mL epidermal growth factor, 1 ng/mL PGE1, 5 × 10-11 M triiodothyronine, 5 × 10-8 M hydrocortisone (Sigma-Aldrich), ITSS media supplement, 1% penicillin/streptomycin, 25 mM HEPES, and 5% FCS (Invitrogen). The cell suspension was then seeded on cell culture Petri dishes and incubated at 37 °C for 2–3 hours to facilitate adherence of contaminating glomeruli. The non-adherent tubules were collected and cultured on collagen-coated Petri dishes (BD Biosciences) in K1 medium. Expression of the epithelial cell marker cytokeratin was verified by immunofluorescent staining with an anti-cytokeratin antibody (Sigma-Aldrich). Cells were >95% cytokeratin positive. Experiments were commenced after cells had reached 80% confluence.

### High glucose stimulation of podocytes or TEC *in vitro*

Cultured podocytes or TECs at 80% confluence were rinsed and incubated with serum-free DMEM/F12 medium with 0.5% ITSS supplement for podocytes, or serum free K1 medium for TECs for 24 hours. The cells were exposed to 30 mM D-glucose (Invitrogen) or mannitol (5.5 mM glucose + 24.5 mM mannitol) with or without 100 ng/ml rIL-17A (R&D Systems, Inc.MN, USA) in fresh 0.5% ITSS-supplemented DMEM/F12 medium for podocytes or K1 medium for TECs for 24 hours. After stimulation, the cells were harvested for PCR assay.

### Statistical analysis

All data are presented as mean ± SD. Data between two groups were analysed by *t-*tests, and multiple groups were compared using one- or two-way ANOVA with *post-hoc* Bonferroni’s correction (Graph Pad Prism 6 software, San Diego, CA). A *p* value less than 0.05 was considered statistically significant.

## Data Availability

All data generated during and/or analysed during the current study are available from the corresponding author upon reasonable request.
